# Hemolytic crisis in a G6PD-deficient infant after ingestion of pumpkin

**DOI:** 10.1186/1824-7288-40-71

**Published:** 2014-07-21

**Authors:** Gian Vincenzo Zuccotti, Francesca Redaelli, Valentina Gualdi, Valeria Rizzi, Chiara Mameli, Dario Dilillo, Valentina Fabiano

**Affiliations:** 1 Department of Pediatrics, Luigi Sacco Hospital, University of Milan, Via G.B. Grassi, 74, 20157 Milan, Italy; 2 Parco Tecnologico Padano, Via Einstein, Loc Cascina Codazza, 26900 Lodi, MI, Italy

**Keywords:** Glucose-6-phosphate dehydrogenase, Favism, Children, Hemolysis, Pumpkin

## Abstract

A 8 month-old infant presented with acute onset of severe jaundice, anemia requiring transfusion and Glucose-6-Phosphate Dehydrogenase deficiency. The infant did not take drugs, he did not consume fava beans, but fava beans DNA was found on pumpkin he consumed the day before jaundice onset. This is the first case of hemolysis triggered by ingestion of food cross-contaminated with fava beans.

## 

Glucose-6-Phosphate Dehydrogenase (G6PD) deficiency is the most common human enzymatic deficiency, caused by a mutation in the G6PD gene, localized on X-chromosome. Subjects affected by G6PD deficiency are prone to experience hemolytic crisis following oxidative stresses, caused by infections, administration of some drugs and ingestion of fava beans; for that reason, the disease is also commonly called favism [1]. Favism is fairly common in Italy; especially in Sardinia [2].

A male 8-month old infant, presented at our Emergency Room (ER) in February 2014 for jaundice noticed for nearly 24 hours. He was born at term, healthy, after an uncomplicated pregnancy. He was breastfed until 6 months of age; weaning was started from 4 months of age, without any relevant problem. Family history was unremarkable. The days before jaundice onset he was healthy, did not present infections or fever, did not take any drug or supplement and did not consume either food containing fava beans or legumes not previously already eaten.

At admission to ER, the child was in good condition. His medical examination was normal except for a remarkably yellowish skin. Blood examinations showed unconjugated hyperbilirubinemia (total bilirubin: 10.6 mg/dL; unconjugated bilirubin: 10.3 mg/dL), moderate anemia (7.6 g/dL, Ht: 23%), reduction of red cells count (2.500.000/mm3), anisocytosis and reticulocytosis (3.7%). Routine biochemical and urinary parameters and an abdomen ultrasound were normal. A second blood examination, 12 hours later, showed severe anemia (Hb: 5.6 g/dL, Ht 17.7%, reticulocytosis 5.9%), increased lactodehydrogenase value (739 U/L), negative direct Coombs test. A red cell transfusion was performed and well-tolerated. Serologies for common infectious pathogens (Epstein Barr Virus, Parvovirus, Citomegalovirus, Hepatitis A, B and C viruses, Toxoplasma, Mycoplasma) were negative. Haemoglobin electrophoresis was normal. Occult blood test was negative. Dosage of G6PD showed en enzymatic deficiency (16 U/1012 RBC; reference ranges 146–376 U/1012 RBC) and a diagnosis of favism was made. A precise drug and food anamnesis was made, searching for a precipitating factor for hemolysis: the day before jaundice onset, the infant ate pumpkin for the first time. We hypothesized that the pumpkin may have been cross-contaminated with fava beans.

## Findings

**Figure 1:**
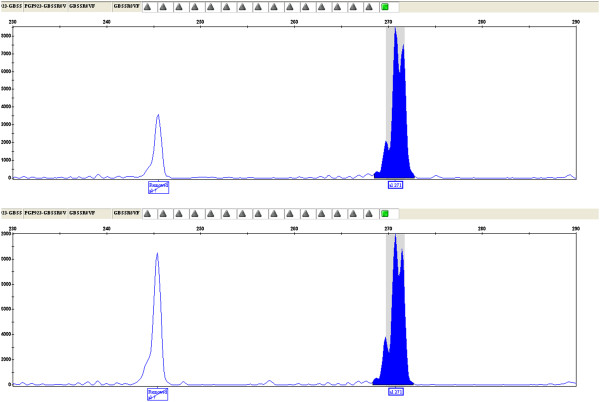
Electrophoretic profile of Vicia faba microsatellite molecular marker GBSSR-VF-8.

**Figure 2:**
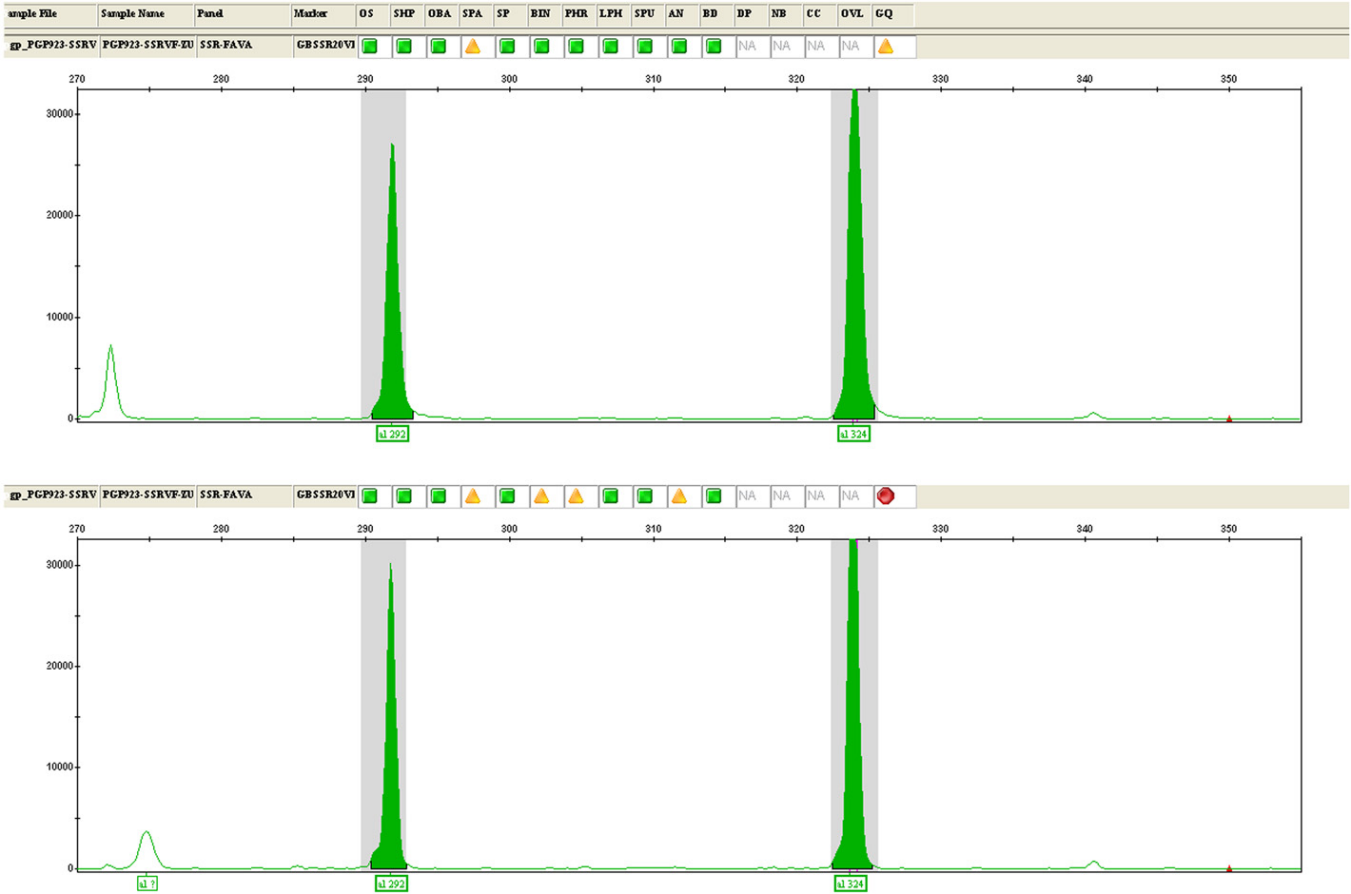
Electrophoretic profile of Vicia faba microsatellite molecular marker GBSSR-VF-20.

A sample of frozen pumpkin (Cucurbita maxima), those consumed by the child, was sent to the Laboratory of the Genomics Platform - Parco Tecnologico Padano (PGP), an Italian reference laboratory for molecular analysis in the field of genomics applied to the agri-food sector. A three-step analysis was performed. First, DNA was extracted from the surface of the pumpkin sample using a commercial column kit (QIAMP Fast DNA Stool Qiagen®). Secondly, a test for DNA amplificability (qualitative-PCR for the trnL gene sequences in the chloroplast of the plant) showed that the obtained DNA was perfectly amplifiable, without evidence of degradation or inhibition; sequencing of the amplified product showed a 100% similarity to Cucurbita maxima trnL gene reference sequences (NCBI). Then, soybean (Glicyne max), pea (Pisum sativum) and fava bean (Vicia fava) DNA were searched for in the pumpkin extracted DNA. Signal amplification of specific markers for soybean (Lectine gene) and pea (SSR markers) were not found, on the contrary, the amplification products of fava bean markers were detected in the analyzed sample. To verify the presence of fava bean DNA, the PGP team analyzed microsatellite molecular markers (SSR - simple sequence repeat). These markers are a stretch of repeated DNA, made up of a variable number of tandem repeats of a limited number of nucleotides. A set of four Vicia faba specific SSR markers (GBSSR-VF-8, GBSSR-VF-19, GBSSR-VF-20, GBSSR-VF-52) was used [3] and two (GBSSR-VF-8 and GBSSR-VF-20) of them showed amplification products with the size expected for Vicia faba on the DNA extracted from the surface of the pumpkin (Figures 1 and 2). We hyphotesized a case of cross-contamination between pumpkin and fava bean. The ingestion of substances present in the fava beans, pyrimidine, vicine and convicine’s beta-glucosides, can trigger hemolytic crisis with variably severe clinical manifestations. The combination of some factors, such as residual enzymatic activity, the metabolic characteristics of the individual, the general health state or the amount of fava beans ingested, may have a relevant role to determine the severity of the symptoms [1]. Most of the episodes of favism has been reported in individuals with severe enzymatic deficiency [4]. Children with G6PD deficiency seem to be more prone to hemolytic crisis triggered by fava beans’ ingestion respect to adults; this is possibly due to the relatively greater exposure for the lower body surface. Fresh fava beans are more harmful than those treated. Beta-glucosides are more concentrated in less mature fava beans, whose ingestion may be correlated to more severe symptoms [5].

To our knowledge, this is the first case of severe hemolytic crisis triggered by ingestion of a fava beans cross-contaminated food. It underlines the importance of considering possible cross-contamination between foods which grow in the same fields or are treated along the same food supply chain. In G6PD deficient subjects, whose enzymatic residual activity is very low or in pediatric patients, cross-contamination of foods with fava beans may possibly represent a risk factor for hemolytic crisis even if a small quantity of contaminated food is ingested. A clear declaration of the possible cross-contamination with fava beans should be reported on product labels, at least for those foods for which it is more likely to occur.

## Consent

Written informed consent was obtained from the patient’s parents for the publication of this report.

## Abbreviations

ER: Emergency room; G6PD: Glucose-6Phosphate-Dehydrogenase.

## Competing interest

The authors declare that they have no competing interests.

## Authors’ contributions

VF, FR, CM, DD and GVZ have written the first draft of the paper, have given final approval of the version to be published; and agree to be accountable for all aspects of the work in ensuring that questions related to the accuracy or integrity of any part of the work are appropriately investigated and resolved; Valentina Gualdi and Valeria Rizzi performed DNA analysis, have given final approval of the version to be published; and agree to be accountable for all aspects of the work in ensuring that questions related to the accuracy or integrity of any part of the work are appropriately investigated and resolved. All authors read and approved the final manuscript.
